# Human myoelectric spatial patterns differ among lower limb muscles and locomotion speeds

**DOI:** 10.14814/phy2.14652

**Published:** 2020-12-05

**Authors:** Bryan R. Schlink, Andrew D. Nordin, Daniel P. Ferris

**Affiliations:** ^1^ J. Crayton Pruitt Family Department of Biomedical Engineering University of Florida Gainesville FL USA

**Keywords:** high‐density electromyography, lower limb, running, spatial activation, walking

## Abstract

The spatial distribution of myoelectric activity within lower limb muscles is often nonuniform and can change during different stationary tasks. Recent studies using high‐density electromyography (EMG) have suggested that spatial muscle activity may also differ among muscles during locomotion, but contrasting electrode array sizes and experimental designs have limited cross‐study comparisons. Here, we sought to determine if spatial EMG patterns differ among lower limb muscles and locomotion speeds. We recorded high‐density EMG from the vastus medialis, tibialis anterior, biceps femoris, medial gastrocnemius, and lateral gastrocnemius muscles of 11 healthy subjects while they walked (1.2 and 1.6 m/s) and ran (2.0, 3.0, 4.0, and 5.0 m/s) on a treadmill. To overcome the detrimental effects of cable, electrode, and soft tissue movements on high‐density EMG signal quality during locomotion, we applied multivariate signal cleaning methods. From these data, we computed the spatial entropy and center of gravity from the total myoelectric activity within each recording array during the stance or swing phases of the gait cycle. We found heterogeneous spatial EMG patterns evidenced by contrasting spatial entropy among lower limb muscles. As locomotion speed increased, mean entropy values decreased in four of the five recorded muscles, indicating that EMG signal amplitudes were more spatially heterogeneous, or localized, at faster speeds. The EMG center of gravity location also shifted in multiple muscles as locomotion speed increased. Contrasting myoelectric spatial distributions among muscles likely reflect differences in muscle architecture, but increasingly localized activity and spatial shifts in the center of gravity location at faster locomotion speeds could be influenced by preferential recruitment of faster motor units under greater loads.

## Graphical abstract

Dynamic high‐density electromyography (EMG) recordings revealed heterogeneous spatial myoelectric patterns in lower limb muscles during locomotion. Spatial EMG activity became more localized as locomotion speed increased, possibly due to preferential activation of specific motor units within each muscle.

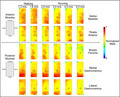

## 1 INTRODUCTION

Human locomotion is a complex task that requires coordinated lower limb muscle activation to accelerate and maintain stability of an individual's center of mass (Pandy & Andriacchi 2010). During walking, the hip and knee extensors are active in early stance to support the leg and trunk, while the ankle plantar flexors activate in midstance to provide further support and forward propulsion (Neptune et al., 2004). The hamstrings activate late in swing phase to decelerate forward motion and prepare for the impact of heel strike (Neptune et al., 2008). Similar mechanics are seen during running, although the ankle plantar flexors activate during late swing (pre‐activation) and early stance (braking; Ishikawa et al., 2007). Together, the actions of these muscles produce a smooth and sustainable cyclic motion (Olree & Vaughan, 1995).

Electrical activity within a muscle is generated through motor unit recruitment, though the spatial distribution of this activity is often non‐uniform (Falla & Gallina, 2020). During isometric contractions, motor units in the vastus medialis muscle take up very little space relative to the entire muscle volume, and pennation of the muscle fibers leads the motor units to produce forces in different directions from each other (Gallina & Vieira, 2015). Pennation angles and heterogeneous muscle fiber force vectors also occur in the medial gastrocnemius (Vieira et al., 2011). Perhaps related to the mechanical heterogeneity, the spatial distribution of muscle activity appears to be inconsistent across tasks and conditions. Healthy individuals shift the location of peak muscle activity in their lower back muscles during repetitive tasks (Falla et al., 2014; Readi et al., 2015), which may act as a mechanism for preventing overuse injuries.

Electrical muscle activity during locomotion is typically measured using surface electromyography (EMG; Winter & Yack, 1987). Bipolar EMG quantifies the average muscle activity in the vicinity of a pair of electrodes (Winter et al., 1994). In contrast, high‐density EMG improves the spatial resolution of standard bipolar EMG by using a grid of electrodes to record from a larger surface area over the target muscle. Recent research using high‐density EMG during locomotion has indicated that different lower limb muscles may have varying patterns of spatial muscle activation patterns (Cronin et al., 2015; Hegyi et al., 2019; Schlink et al., 2020). In a previous study, we demonstrated that channel decomposition and cleaning can effectively remove motion artifacts from high‐density EMG recordings during running (Schlink et al., 2020). We also found contrasting patterns of spatial EMG activity in the tibialis anterior and medial gastrocnemius muscles, though the patterns of EMG activity within each muscle appeared relatively homogeneous across different locomotion speeds. Tibialis anterior EMG activity was more uniform across the muscle with peaks of activity in the proximal and distal regions. The medial gastrocnemius had a much more distinct pattern of activity. The EMG activity with the greatest amplitude occurred at the distal portion of the muscle across all speeds. Cronin et al. observed a similar pattern of spatial EMG activity in the lateral gastrocnemius during walking (Cronin et al., 2015). Hegyi et al. (2019) used a linear array of electrodes to measure biceps femoris EMG activity during running and found peak activity in the middle‐to‐distal regions, although the activation patterns were highly individual‐specific. These studies suggest there is variability in spatial myoelectric activity both across and within individual muscles, although the differing gait speeds and electrode array sizes make it difficult to compare results across studies. No study to date has investigated spatial myoelectric activity during locomotion in more than two muscles, leaving a gap in our understanding of how spatial muscle activation patterns compare among lower limb muscles and how these patterns are influenced by gait speed. Here, we evaluated spatial electrical muscle activity in lower limb muscles crossing the hip, knee, and ankle joints at a range of gait speeds using high‐density EMG. This knowledge can help identify basic spatial myoelectric traits during locomotion and provide a comparative baseline for researchers studying neuromuscular dysfunction or injury.

The goal of this study was to determine if there are similar spatial EMG activity patterns in various lower limb muscles during locomotion, including both walking and running. We recorded high‐density EMG from five muscles of healthy individuals at a range of walking and running speeds on a treadmill. We hypothesized that: (a) EMG activity is spatially heterogeneous across different lower limb muscles during locomotion, and (b) The spatial EMG pattern within a muscle will not change with increasing treadmill speed. This analysis allowed us to comprehensively evaluate the myoelectric patterns among several primary lower limb muscles and gain a better understanding of how neuromuscular recruitment varies during locomotion.

## 2 MATERIALS AND METHODS

### 2.1 Study participants

Eleven healthy volunteers (8 Males, 3 Females; mean age 21 ± 3 years) participated in this study. Every subject had no history of major lower limb injuries or neurological conditions. All procedures were approved by the University of Florida Institutional Review Board, and all subjects provided written informed consent before participating, in accordance with the Declaration of Helsinki.

### 2.2 Experimental protocol

**Figure 1:**
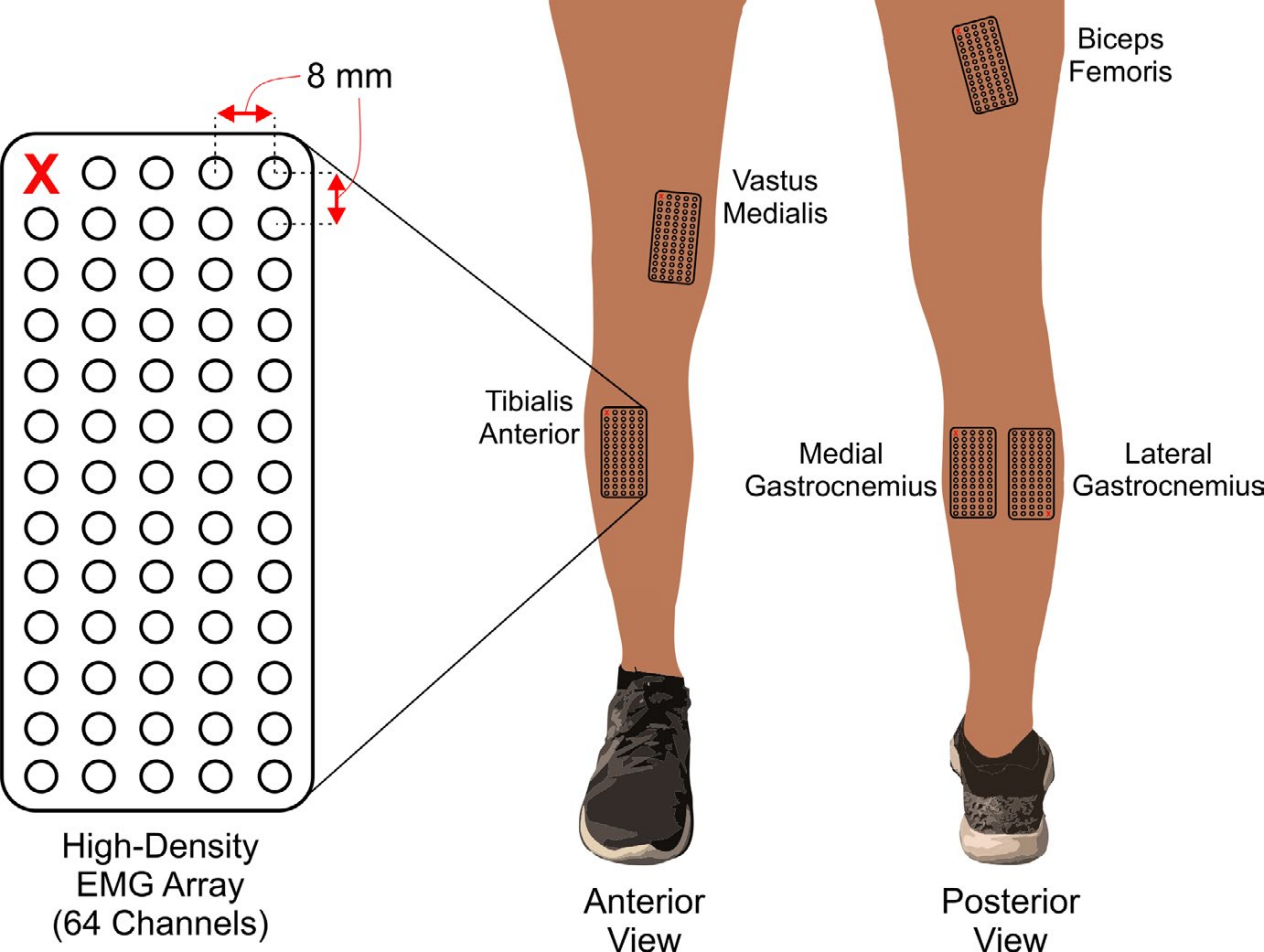
We placed 64‐channel electromyography arrays on five lower limb muscles: biceps femoris, vastus medialis, tibialis anterior, medial gastrocnemius, and lateral gastrocnemius

We measured high‐density EMG from five muscles on each subject's right leg while they walked and ran on an instrumented treadmill. The muscles we measured were the biceps femoris, vastus medialis, tibialis anterior, medial gastrocnemius, and lateral gastrocnemius (Figure 1). Prior to attaching the electrodes, we located the boundaries of each muscle using ultrasonography (ArtUS EXT‐1H, Telemed) to ensure that each EMG array was properly placed on the target muscle. We shaved the hair and cleaned the skin over each muscle using abrasive paste and alcohol swabs.

The EMG array (OT Bioelettronica) consisted of 64 electrodes arranged into 13 rows and 5 columns with an interelectrode spacing of 8 mm. We aligned the longitudinal columns of the array parallel to the muscle longitudinal axis for all muscles except for the vastus medialis. In this instance, we aligned the electrode array nearly vertically so that the entire array was positioned over the muscle belly. To closely resemble electrode placement of bipolar EMG sensors (Hermens et al., 2000) and account for different muscle sizes across subjects, we positioned the center of each electrode array with the midline of the belly of each muscle. Each electrode array was connected to a small adapter box and long ribbon cable to transmit data to the main amplifier. As a result, we were limited on physical space and forced to record EMG data from the five target muscles in two bouts. In the first bout, we recorded data from the vastus medialis, tibialis anterior, and medial gastrocnemius muscles. In the second bout, we recorded EMG data from the biceps femoris and lateral gastrocnemius muscles. The approximate delay in between bouts was 20 min, during which subjects sat and rested.

In each bout, subjects walked and ran on an instrumented treadmill (Bertec FIT, Bertec Corporation) at six different speeds: 1.2 and 1.6 m/s (walking); 2.0, 3.0, 4.0, and 5.0 m/s (running). We recorded data for a duration of 20 strides per leg at each speed. We allowed subjects to take breaks throughout the experiment so they did not fatigue at any point. We randomized the order of speeds for each subject, and that order was the same for each bout that a subject completed.

We recorded 64‐channel EMG monopolar data at a sampling frequency of 2048 Hz with an online bandpass filter of 10–500 Hz. We visually inspected all data after each collection and excluded data from any muscle or trial that contained large amounts of noisy channels or data loss (>16 monopolar channels). Taking into account all subjects, muscles, and speeds, we recorded a total of 330 trials and rejected only three trials due to excessive noise. We recorded ground reaction forces (1,000 Hz) from the instrumented treadmill to determine when ground contact and toe off of the right leg occurred.

### 2.3 Data processing and analysis

To eliminate the influence of noise contamination caused by movement of the recording electrodes, cabling, and lower limb soft tissues during locomotion, we processed the high‐density EMG data using canonical correlation analysis to decompose and clean the channel data from each EMG array (Schlink et al., 2020; Exemplar differential channel cleaning example shown in Figure S1). We downsampled the 64‐channel monopolar EMG data from each array to match the sampling frequency of the force plate data (1,000 Hz). After decomposing the time series high‐density EMG data into canonical components, we performed Fast Fourier Transform to identify canonical components with outlier spectral statistics based on their skewness, kurtosis, or standard deviation within the relevant frequency range (0–500 Hz). We then performed spectral noise cancellation on frequencies in these components that met specific criteria (frequencies > 10 times or <2 times the median spectral amplitude) using methods from Nordin et al. (2018, 2020). Once all components were cleaned, we performed the inverse Fast Fourier Transform and reconstructed the monopolar EMG data using the cleaned components. We converted the 64 clean monopolar channels into 59 differential channels along the longitudinal axis of the EMG array and repeated the same statistical and spectral processing steps to clean the differential channel data. Finally, we high‐pass filtered the differential channels using a 4th‐order high‐pass Butterworth filter with a cutoff frequency of 20 Hz and no lag. In some cases, EMG channels still had poor signal quality due to excessive baseline noise or poor skin contact. We used objective channel exclusion criteria based on statistical characteristics (Gwin et al., 2010, 2011) to remove these channels from further analysis.

We low‐pass filtered the ground reaction force data at 40 Hz (4th order Butterworth filter) to determine ground contact and toe‐off timings of each stride. We defined stance phase to begin when the ground reaction force exceeded 30 N (Havens & Sigward, 2015). Stance phase termination occurred when the force no longer exceeded 30 N (i.e., toe‐off). We used these timings to create epochs of the EMG channel data from the primary phase of the gait cycle that each muscle is active (stance: vastus medialis, medial/lateral gastrocnemii; swing: biceps femoris, tibialis anterior). We temporally normalized the durations of stance and swing to 101 data points (0%–100% of each phase). By temporally averaging the high‐density EMG differential signals from the recording array that overlaid each lower extremity muscle, we visualized the muscle activation pattern at each locomotion speed. We calculated the average root mean square (RMS) value in the target gait cycle phase for all 59 differential channels of the EMG array and averaged these values across the 20 strides that were recorded. This allowed us to produce spatial amplitude maps of EMG activity for each muscle. We normalized these spatial maps within each subject two different ways: (a) to the peak RMS value across all walking and running speeds, and (b) to the peak RMS value within each walking and running speed. We averaged the results of each normalization method across all subjects to produce final EMG spatial amplitude maps for all muscles and speeds. Every spatial map was interpolated by a factor of 8 for a smoother visual output of the EMG activity, but we only used the original 59 RMS values during the statistical analysis.

To assess the homogeneity of spatial EMG amplitude patterns, we calculated the modified entropy of the RMS values within each EMG array and the location of the center of gravity (Farina et al., 2008; Madeleine et al., 2006). In this context, entropy measures the level of myoelectric spatial heterogeneity (uniformity) measured throughout the electrode array. Higher entropy values indicate greater myoelectric spatial homogeneity (less localized). Lower entropy values indicate lesser myoelectric spatial homogeneity (more localized). Maximum spatial EMG entropy occurs if all RMS values from the EMG array are equal. The center of gravity was calculated by weighing each electrode's RMS value and position relative to the sum of RMS values from all 59 electrodes. The center of gravity indicates the barycenter of EMG amplitude. We measured changes in the center of gravity in each direction individually (*x*‐direction: lateral‐medial; *y*‐direction: proximal‐distal).

### 2.4 Statistical analysis

Prior to running statistical analyses, we confirmed that all variables were normally distributed, and we applied a Greenhouse‐Geisser correction to account for violations in sphericity. We performed a two‐way ANOVA (*α* = 0.05) to test for statistical differences in mean entropy values, with muscle and speed as between‐subjects factors. Since we measured both anterior and posterior muscles of the lower limb, there is no straightforward coordinate system nor spatial normalization method to easily compare changes in EMG amplitude center of gravity position among muscles. Therefore, we analyzed each muscle individually using separate one‐way repeated measures ANOVAs to test for statistical differences in the *x* and *y* center of gravity coordinates of each muscle across all locomotion speeds (10 total ANOVAs: *x*‐ and *y*‐position for five muscles). We completed Bonferroni adjusted pairwise comparisons when necessary for each ANOVA.

## 3 RESULTS

**Figure 2:**
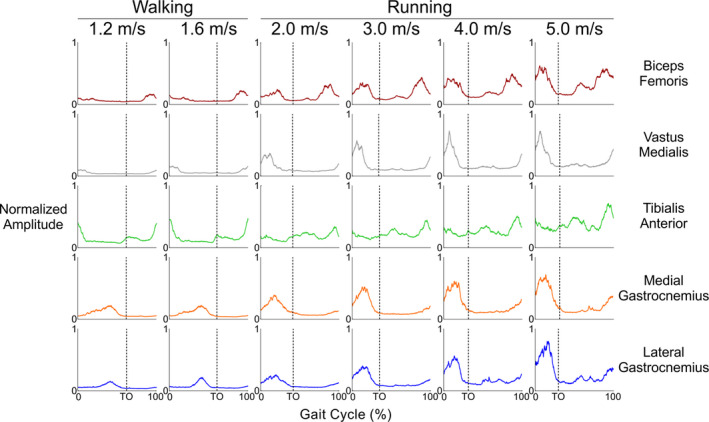
Group average muscle amplitude patterns across the entire gait cycle for all five lower limb muscles (rows) and locomotion speeds (columns). Time series activation patterns were aggregated among differential electromyography (EMG) recordings from the entire high‐density EMG array overlaying each muscle. The EMG channel amplitudes from each muscle were normalized within each subject to the maximum value across all speeds (*y*‐axis). Strides begin and end at ground contact for the right leg. Vertical dashed lines indicate when toe‐off (TO) occurred for the right leg

The average EMG amplitude patterns across the entire high‐density array followed the same patterns typically seen when recorded with traditional bipolar EMG electrodes (Figure 2; Gazendam & Hof, 2007; Winter & Yack, 1987). Muscle activations predominantly occurred during late swing phase and throughout stance phase in the lower limb extensors (vastus medialis, medial and lateral gastrocnemii). Flexor muscle activations predominantly occurred during limb swing, with co‐contraction immediately prior to and during stance, particularly during running (biceps femoris, tibialis anterior). Qualitatively, muscle activation peak amplitude tended to increase with speed, and peak EMG amplitudes occurred at the fastest speed for all five lower limb muscles that we measured.

**Figure 3:**
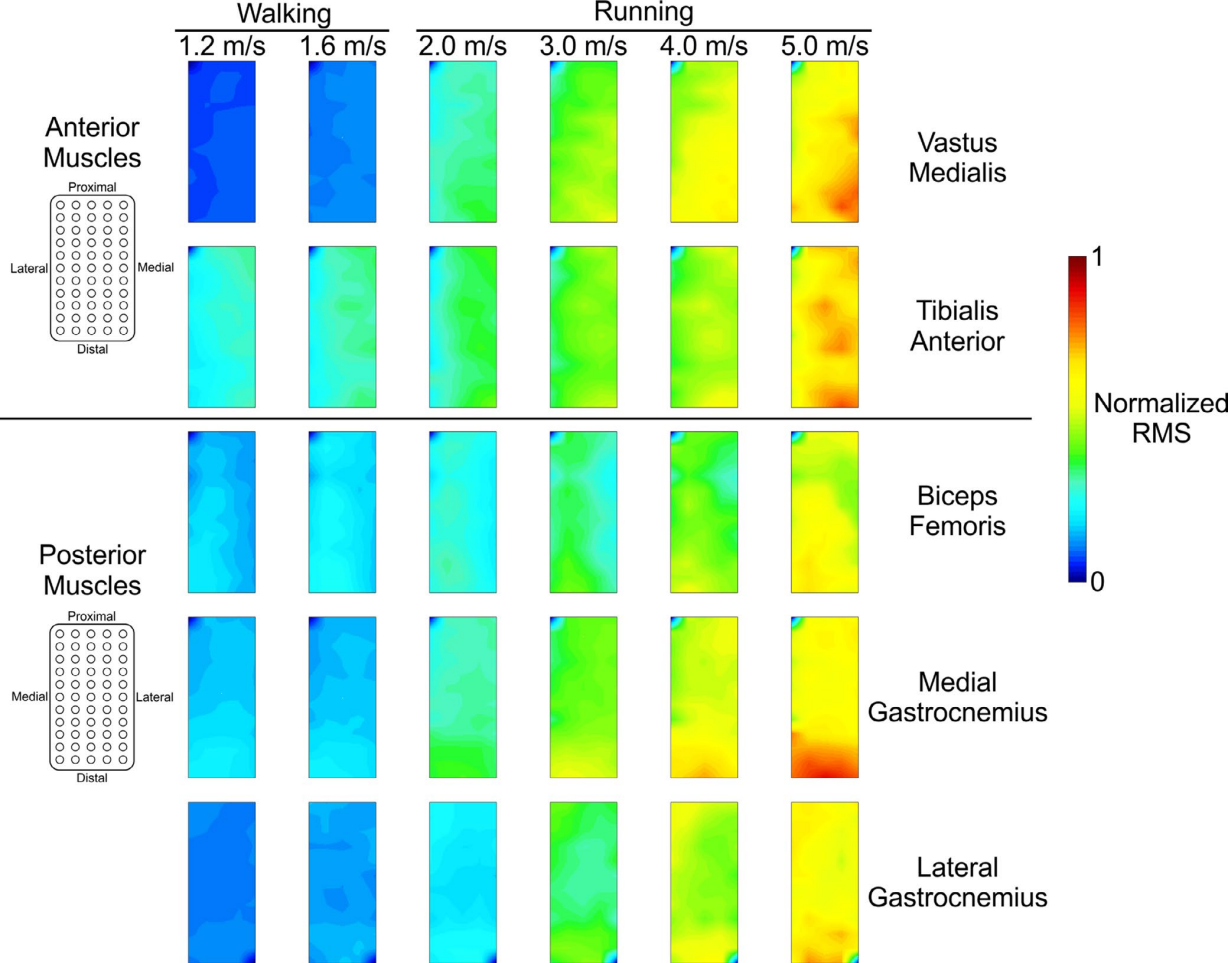
Group average electromyography (EMG) spatial amplitude maps for all five muscles (rows) at each walking and running speed (columns). Data from each muscle were normalized within each subject to the maximum root mean square (RMS) value across all speeds. The EMG profiles for the biceps femoris and tibialis anterior EMG patterns were averaged over the swing phase. The vastus medialis, medial gastrocnemius, and lateral gastrocnemius EMG profiles were averaged over the stance phase

The spatial EMG amplitude patterns differed among muscles (Figure 3). We visually observed increased EMG activity over the central‐to‐distal regions of the biceps femoris and vastus medialis muscles. Tibialis anterior EMG activity was more uniformly distributed across the entire muscle, and the change in EMG‐RMS amplitude across speeds for this muscle was lesser than the other muscles. The greatest EMG‐RMS activity in the medial and lateral gastrocnemii during stance phase was at the distal portion of the muscle underlying the electrode array. In all muscles, we observed the greatest average EMG‐RMS amplitude in the fastest trial (5.0 m/s), and all muscles displayed a trend of increased RMS activity with increasing treadmill speed, in alignment with the temporal activation patterns in Figure 2.

**Figure 4:**
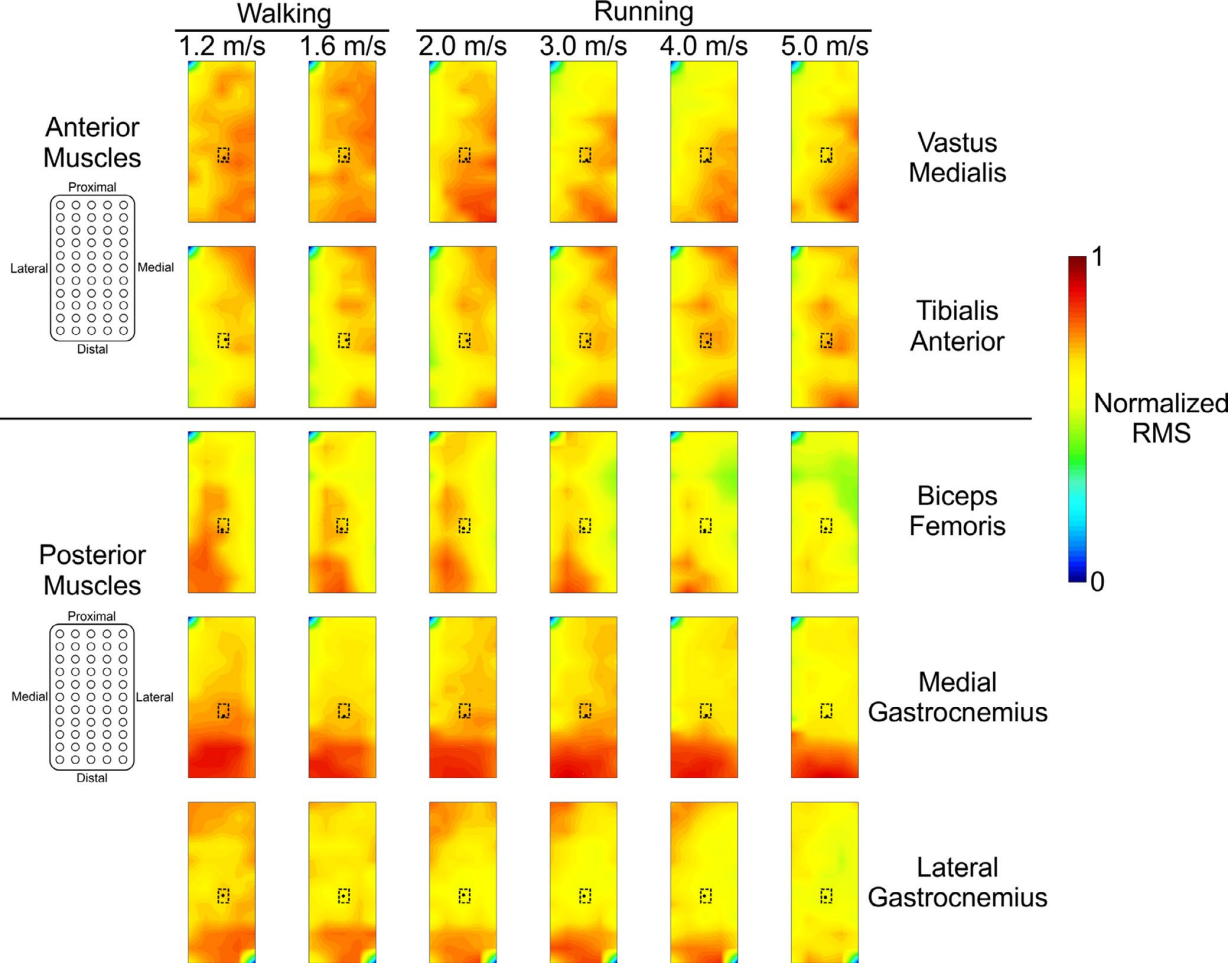
Group average electromyography (EMG) spatial amplitude maps for all five muscles (rows) at each walking and running speed (columns). Data from each muscle were normalized within each subject to the maximum root mean square (RMS) value within each speed. The EMG profiles for the biceps femoris and tibialis anterior EMG patterns were averaged over the swing phase. The vastus medialis, medial gastrocnemius, and lateral gastrocnemius EMG profiles were averaged over the stance phase. The area bordered by dashed lines indicate the area in which the centers of gravity in Figure 6 are plotted. The black dot on each map indicates the center of gravity for that condition

Qualitatively, the spatial distribution of EMG activity within each muscle was relatively consistent across treadmill speeds (Figure 4). Spatial EMG homogeneity tended to decrease in the biceps femoris muscle as speed increased. The EMG activity in the vastus medialis, medial gastrocnemius, and lateral gastrocnemius muscles was more evenly distributed at lower speeds and became more localized as speed increased (greater heterogeneity). The tibialis anterior muscle had greater proximal EMG amplitudes at lower speeds, whereas amplitudes in the distal portion of the muscle increased with locomotion speed. All muscles had centers of gravity located near the center of the electrode array, regardless of speed (Figure 4, black circles). Quantitative differences among spatial centers of gravity are described in a subsequent paragraph.

**Figure 5:**
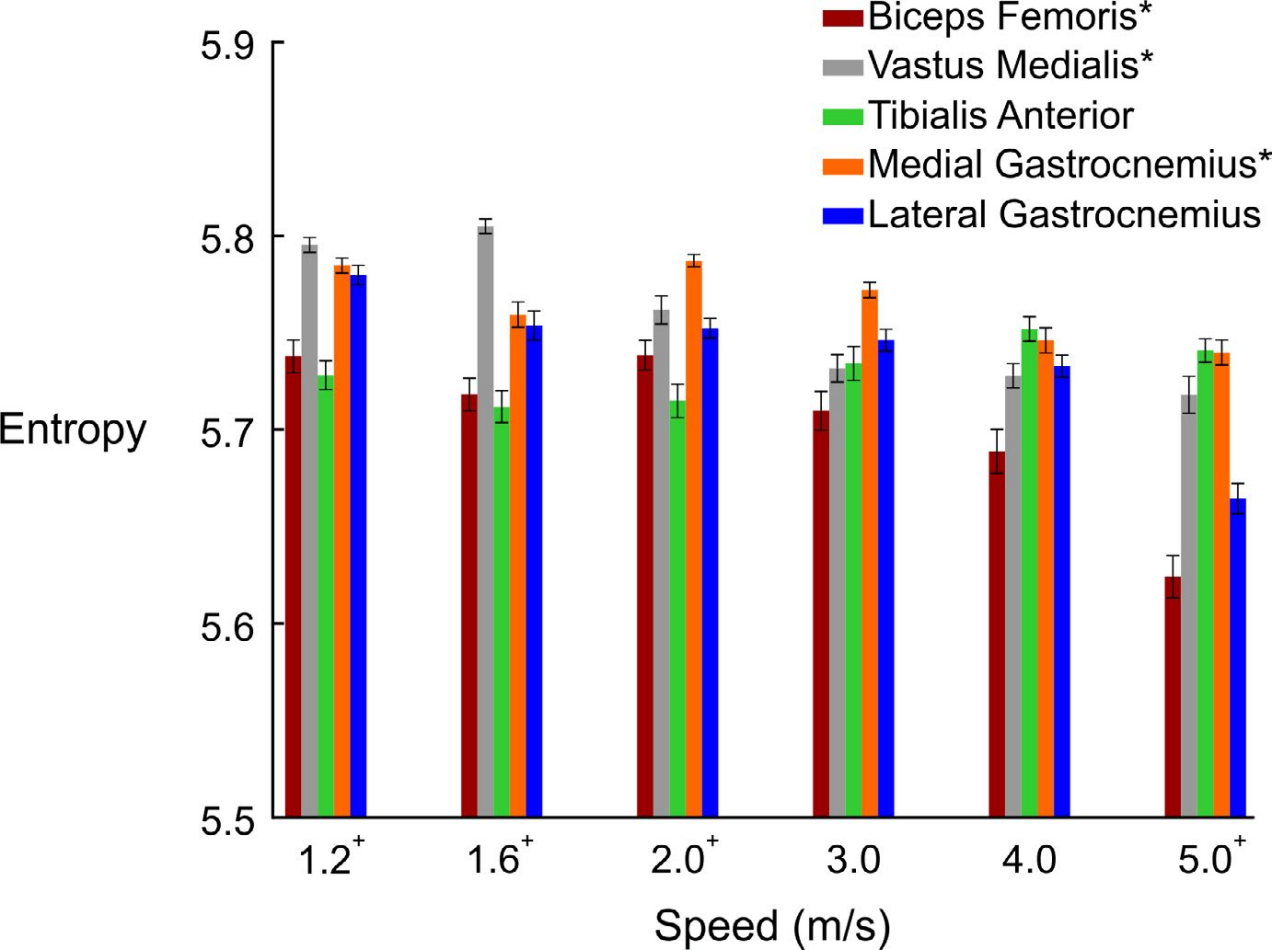
Mean entropy values for each muscle over their target phase of the gait cycle (stance: medial & lateral gastrocnemii, vastus medialis; swing: tibialis anterior, biceps femoris) at all walking and running speeds. Error bars represent standard error

Based on the calculation of entropy of the high‐density EMG array, there was no significant interaction between lower limb muscles and locomotion speeds (*F*_20,297_ = 1.1, *p* = .325). Spatial muscle entropy decreased (more heterogeneity or localized activity) as locomotion speed increased (*F*_5,297_ = 4.8, *p* < .001; Figure 5). The lowest entropy value among lower limb muscles occurred in the fastest (5.0 m/s) running trial, relative to 1.2, 1.6, and 2.0 m/s (*p* < .009 for all). There was also a significant main effect for lower limb muscle (*F*_4,297_ = 6.1, *p* < .001), with biceps femoris spatial muscle entropy less than both the medial gastrocnemius and vastus medialis muscle entropies (*p* < .002 for both).

**Figure 6:**
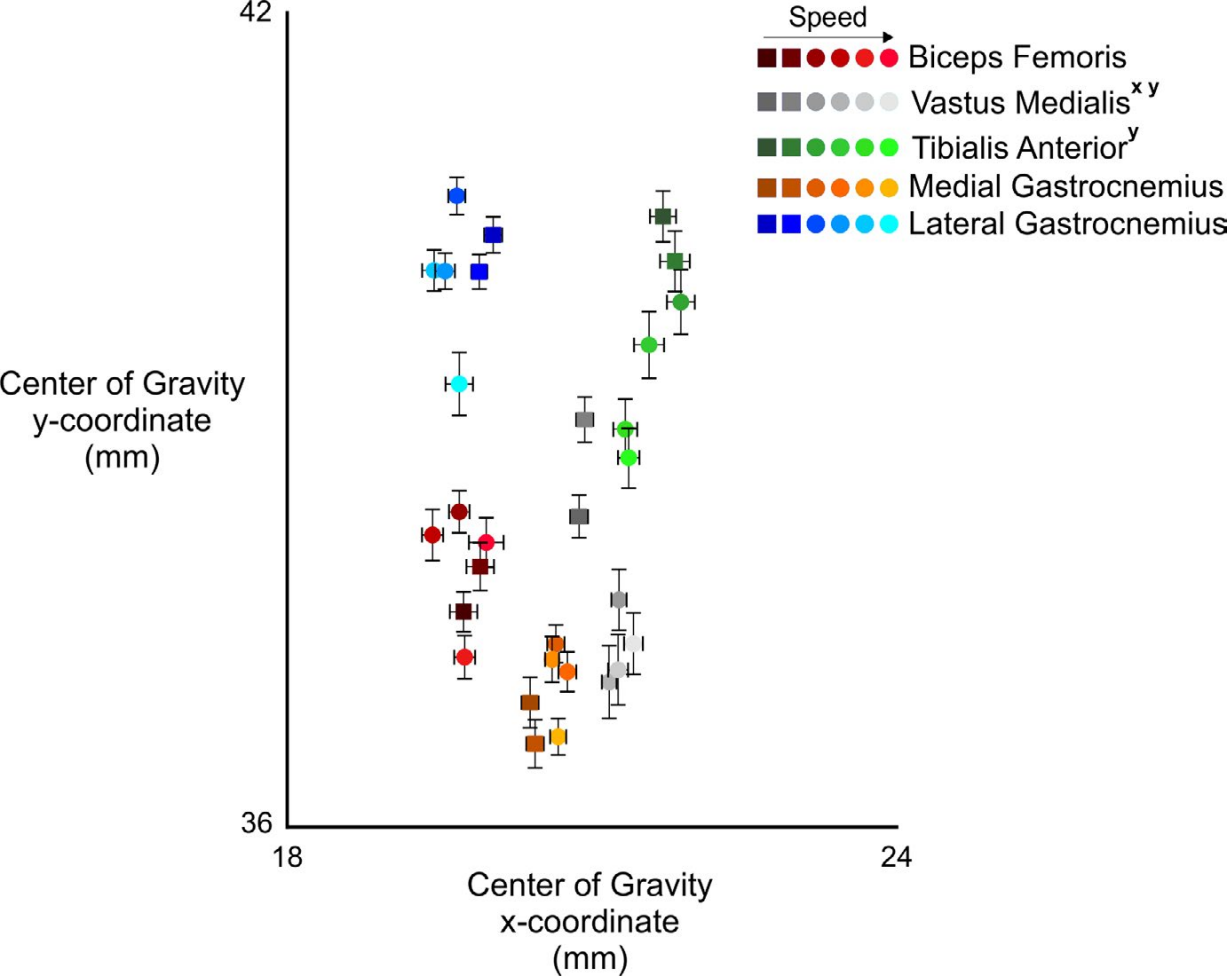
Center of gravity coordinates (*x* and *y*) for each muscle over their target phase of the gait cycle (stance: medial & lateral gastrocnemii, vastus medialis; swing: tibialis anterior, biceps femoris) at all walking and running speeds. The area shown is defined by the dashed‐line box in Figure 4. The colors for each muscle become lighter as speed increases. Walking speeds are denoted by squares and running speeds are denoted by circles. Error bars represent standard error. Significant main effects of speed within each muscle (*p* < .05) for the *x*‐ and *y*‐coordinates are denoted in the figure legend by *^x^* and *^y^*, respectively

The *x*‐coordinate of the spatial muscle center of gravity location differed only in the vastus medialis muscle across speeds (*F*_3.0,26.6_ = 4.6, *p* = .011; Figure 6). Pairwise comparisons showed that faster locomotion speeds caused a medial shift in vastus medialis activity (*p* < .036). We did not find any significant differences in the center of gravity *x*‐coordinate in the other four muscles (*p *> .079 for all). The *y*‐coordinate of the spatial muscle activity center of gravity differed among locomotion speeds in both the tibialis anterior (*F*_3.1,28.2_ = 5.1, *p* = .005) and vastus medialis muscles (*F*_2.5,22.6_ = 4.6, *p* = .015). Pairwise comparisons in the tibialis anterior and vastus medialis muscles showed that the center of gravity during fast running (5.0 m/s) was significantly more distal than walking (*p* < .047). There were no differences in the *y*‐coordinate center of gravity in the biceps femoris, lateral gastrocnemius, and medial gastrocnemius muscles (*p *> .320 for all).

## 4 DISCUSSION

We measured high‐density EMG from five lower limb muscles at a range of walking and running speeds and found heterogeneous spatial myoelectric activity among the muscles. The greatest EMG amplitude in the gastrocnemii was located in the distal portion of the muscle, while the biceps femoris, vastus medialis, and tibialis anterior muscles had more evenly distributed patterns of EMG activity. The lack of homogeneity among muscles was reflected in the significant differences we observed in the average entropy values. Within each muscle, there was some variation in the EMG spatial activity across locomotion speeds, indicating that peak EMG amplitude was more spatially localized, or heterogeneous, at faster speeds. The center of gravity location shifted distally in the tibialis anterior and vastus medialis muscles as locomotion speed increased, while no significant shifts occurred in the remaining three muscles. Together, our entropy and center of gravity analyses suggest that although the centroid of the EMG‐RMS values remained relatively unchanged, changes occurred in the spatial distribution of the myoelectric activity.

We observed the most distinct pattern of electrical muscle activity in the medial gastrocnemius muscle, where the greatest amplitude was more highly concentrated at the distal portion of the muscle across all locomotion speeds (Figure 4). Cronin et al. observed a similar EMG spatial pattern in the lateral gastrocnemius during walking and suggested that functional compartments of motor units may cause certain areas of the muscle to have a higher amplitude than others (Cronin et al., 2015). We corroborate this distally‐weighted pattern in the lateral gastrocnemius muscle amplitude, and we also observed portions of increased activity in the proximal region. The lateral gastrocnemius, biceps femoris and vastus medialis muscles each had more uniform distributions of EMG activity at the slowest speeds and became increasingly more focused as running speed increased. Motor unit recruitment strategies may have changed to accommodate the activation and force output required at faster running speeds, preferentially enlisting faster motor units (Lee et al., 2013; Wakeling, 2004). Tibialis anterior EMG amplitude was greatest in the proximal portion during walking, along with smaller regions of increased amplitude in the middle portion of the electrode array. At faster running speeds, EMG amplitude was greatest in the distal portion of the electrode array, consistent with previous results (Schlink et al., 2020). Peak EMG amplitude in the biceps femoris occurred in the medial‐to‐distal portions of the muscle underlying the EMG array, and we did not find any significant change in the center of gravity *x*‐ or *y*‐coordinates. Hegyi et al. found similar results across a range of running speeds (Hegyi et al., 2019). Hamstring injuries often occur in the proximal region of the biceps femoris (Fiorentino & Blemker, 2014; Hegyi et al., 2019; Silder et al., 2010), so lower EMG‐RMS activity in this region during running may represent a neuromuscular adaptation to minimize overuse of these muscle fibers and avoid injury.

Contrasting spatial heterogeneity among lower extremity myoelectric activity may be due to several factors. Regional differences in spatial amplitude may result from preferential activation of specific motor unit groups within a muscle. Localized patterns of EMG activity have been found in the medial gastrocnemius during standing (Hodson‐Tole et al., 2013) and in the vastus medialis during isometric knee extensions (Gallina et al., 2018). The relative mechanical capabilities of the different muscles likely influence both the amplitude and location of EMG activity. The gastrocnemii typically have a higher relative proportion of fast‐twitch fibers (Johnson et al., 1973), and there may be a larger grouping of these fibers at the distal portion of the muscle that increasingly act together as speed increases. Conversely, the tibialis anterior muscle has a much lower percentage of fast‐twitch fibers, and previous research has shown lower regional grouping of these fibers (Johnson et al., 1973). Changes in the muscle architecture throughout the gait cycle can also create variations in spatial myoelectric patterns. Vieira et al. showed that architectural changes in the tibialis anterior muscle affected the surface EMG amplitude (Vieira et al., 2017). Differences in pennation among the muscles we analyzed and locations of the superficial aponeuroses may further affect this relationship. Fat acts as an isotropic filter, so thicker layers of subcutaneous tissue lead to a greater spread in the action potentials recorded at the skin surface (Farina et al., 2002; Vieira et al., 2010). The muscles of the shank are typically covered by thinner layers of adipose tissue than the muscles on the hamstrings and quadriceps (Ishida et al., 1995), which may partially explain the distinct pattern observed in the gastrocnemii and the less localized pattern in the biceps femoris and vastus medialis muscles. Coverage of the muscle belly by superficial aponeuroses may also affect the surface EMG amplitude (Vieira et al., 2011). Future research should examine the relative contributions from each of these factors, as the cause of variations in lower limb spatial EMG amplitudes during locomotion may be multifactorial. Ultrasonic recordings of lower limb muscles during locomotion can provide direct measurements of changes in muscle architecture throughout the gait cycle (Nuckols et al., 2020). This may be useful to distinguish how neuromuscular recruitment and architectural changes affect regional variations in the spatial EMG patterns.

We observed the lowest entropy values for all muscles except the tibialis anterior during running at 5.0 m/s. Lower entropy values represent a greater level of heterogeneity in the spatial pattern of EMG amplitude. At faster speeds, we observed increased EMG amplitudes in all muscles, which may result from increased motor unit recruitment and firing rates (Moritani & Muro, 1987), as well as a possible increase in synchronization among the active motor units (Yao et al., 2000). Although we did not directly compare myoelectric center of gravity position changes among lower extremity muscles, we observed contrasting directional shifts among muscles with respect to locomotion speed. Medial and distal shifts in vastus medialis peak EMG amplitudes during stance and distal myoelectric shifts in tibialis anterior EMG amplitudes during swing demonstrate the divergent responses of lower limb flexor and extensor muscles with increased gait speed. Though the differences in these centroid changes are on the order of millimeters, they represent much larger changes in the actual anatomical distribution of EMG activity (Elswijk et al., 2008; Falla & Gallina, 2020). We calculated each muscle's EMG center of gravity from all RMS values across the electrode array, though several muscles showed distinct areas of increased EMG amplitude (Figure 4). A method of segmentation (Vieira et al., 2010) may better describe the barycenter of specific clusters of increased EMG amplitudes. We also used absolute shifts in the center of gravity from each subject and muscle rather than adjusting for individual muscle lengths. Contrasting muscle functions, anatomy, and fiber architecture prohibit direct comparisons of absolute EMG amplitude barycenter locations among muscles. Nevertheless, we found statistical differences in the center of gravity locations with increasing locomotion speed in the tibialis anterior and vastus medialis muscles (Figure 6). Taken together, the results from our high‐density EMG center of gravity and entropy analyses suggest that, despite the consistency observed in the barycenter location, the spatial distribution of this activity actually became more heterogeneous as speed increased.

There were limitations in this study. Within each muscle, we converted the original 64 monopolar signals into 59 differential signals along the longitudinal axis of the array. The distal muscle fibers of the vastus medialis are oriented at an angle of approximately 50° with respect to the longitudinal axis of the femur, whereas the proximal fibers have a much smaller pennation angle (Gallina & Vieira, 2015). Others have accounted for this by placing the electrode array over the muscle based on the location of the innervation zone (Gallina et al., 2018), converting the differential along the diagonal axis of the array (Gallina et al., 2013), or placing smaller linear arrays at angles consistent with fiber orientation in different regions of the muscle (Cabral et al., 2018). We compared the results of vastus medialis EMG activity when computing differential signals along the vertical and diagonal axes and found similar patterns of spatial EMG activation (Figure S2). The contraction level and the individual's locomotion speed may also affect the pennation angle. Of course, comparisons of spatial myoelectric patterns among muscles becomes increasingly difficult when using contrasting electrode orientations or computations of differential signals. We also wanted to ensure that all electrodes on the array overlaid the target muscle. We did not control for differences in the thickness of subcutaneous layers among muscles and subjects, which potentially affected the amplitude measured at the surface. However, we normalized EMG data within each muscle and subject. This likely reduced intersubject variability, though it may not have eliminated it completely. Due to the size and physical setup of the recording system, we measured EMG activity while subjects ran on a treadmill in a laboratory setting. It is possible that our results might differ if we performed the same analysis during overground locomotion, although current opinions differ as to whether EMG data recorded in each condition appreciably differ (Baur et al., 2018; Wang et al., 2014; Wank et al., 1998). Future advancements in high‐density EMG technology will allow researchers to become more mobile, and muscle recruitment can then be studied in real‐world environments. Finally, spatial EMG amplitude patterns may be affected by changes in muscle length throughout the gait cycle. Previous research has shown that tendon stretch allows muscles to operate nearly isometrically during steady‐state running (Roberts, 2002), with validation of this concept shown in several individual lower limb muscles during their primary phase of activation during locomotion (Lichtwark et al., 2007; Munsch et al., 2020; Van Hooren & Bosch, 2017). Therefore, changes in muscle length were likely small and did not greatly bias the EMG patterns we recorded.

We chose to focus the analysis of each muscle on the phase of the gait cycle (stance or swing) that each muscle is primarily active. However, none of the muscles we analyzed are exclusively active in one acute gait sub‐phase across our selected locomotion speeds. Investigating smaller time windows or gait sub‐phases (e.g., prelanding, braking, propulsion, etc.) may provide more specific information regarding motor unit activation patterns. Additionally, the manner in which each subject's foot contacts the ground could influence EMG activity in the gastrocnemii, particularly during running. Due to the increased eccentric loading of the gastrocnemius muscle while braking, subjects who run with a forefoot strike pattern have shown increased muscle activation amplitude and muscle fiber force production than those who run with a heel strike pattern (Yong et al., 2020). We did not control for foot strike patterns in this study, and it is possible that regional differences in EMG‐RMS amplitude may exist between these sub‐groups of subjects. Future studies should consider these individual differences and their effects on the different gait sub‐phases with regard to the spatial distribution of EMG activity.

The heterogeneity in the spatial EMG amplitude patterns is likely due to a combination of mechanics, changes in muscle architecture, and neural control, a relationship that can be explored further through musculoskeletal models. These models typically simulate the muscle as one large muscle fiber using average properties of the entire muscle, thereby neglecting localized effects of independent motor units (Wakeling et al., 2012). EMG‐driven musculoskeletal models of human gait use a single bipolar sensor to characterize the force output of the entire muscle (Manal et al., 2012). However, our results demonstrate that this assumption may not be valid during locomotion due to spatial heterogeneity within several muscles. Recent studies have improved the physiological accuracy by developing models that simulated individual muscle fiber and motor unit properties (Sharafi & Blemker, 2010; Wakeling et al., 2012). By incorporating dynamic high‐density EMG measurements, models may be able to better simulate the regional differences in neuromuscular recruitment during locomotion.

We focused our analyses on the spatial EMG patterns in healthy individuals during locomotion and found that increasing locomotion speed leads to more localized, heterogeneous spatial EMG amplitudes. This may be a neuromuscular mechanism to selectively recruit muscle fibers that are more resistant to overuse or avoid regions within a muscle that are more susceptible to injury. It remains unclear how increased muscle loading or fatigue affect the areas of the muscle with the greatest EMG activation. Additionally, these spatial EMG amplitude patterns may change in the presence of pain, fatigue, and/or injury. During repetitive tasks in relatively stationary conditions, participants with chronic pain in the lower back (Falla et al., 2014) and neck (Barbero et al., 2016; Gerdle et al., 2010) have shown altered patterns of myoelectric recruitment compared to healthy participants. Spatial variations in the amplitude and frequency spectrum of the medial gastrocnemius muscle activity also appear in the presence of fatigue from isometric contractions (Gallina et al., 2011). These conditions have yet to be investigated during locomotion. Regional alterations in electrical muscle activity may also be task specific and vary across individuals (Falla & Gallina, 2020), so a broader scope of research is needed to fully describe any potential changes to neuromuscular recruitment. We have demonstrated that high‐density EMG can measure spatial myoelectric activity in lower limb muscles of healthy adults during locomotion.

## AUTHORS' CONTRIBUTIONS

B.R.S., A.D.N., and D.P.F conceived and designed the research; B.R.S. performed the experiments; B.R.S. analyzed the data; B.R.S. and A.D.N. interpreted results of the experiments; B.R.S. prepared the figures; B.R.S. drafted the manuscript; B.R.S., A.D.N., and D.P.F. edited and revised the manuscript; B.R.S., A.D.N., and D.P.F approved final version of the manuscript.

## Supporting information



Fig S1

Fig S2
