# Supplementary figures and images for: Human myoelectric spatial patterns differ among lower limb muscles and locomotion speeds
Source: Physiol Rep. 2020 Dec 5;8(23):e14652. doi: 10.14814/phy2.14652 (PMC7718836; doi:10.14814/phy2.14652)

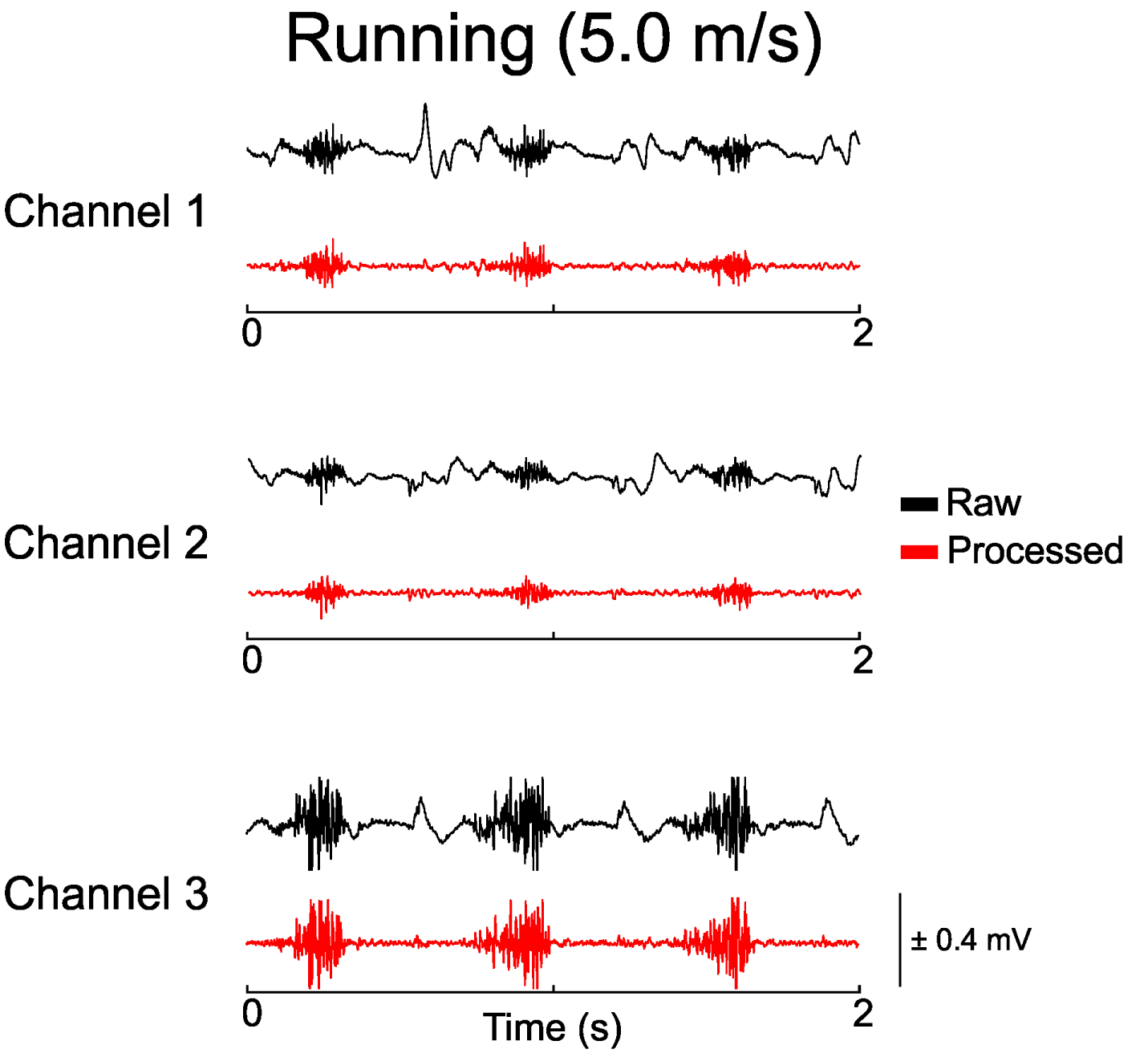

Supplement: Supplementary file 1 — Fig S1 [file PHY2-8-e14652-s001.tif]

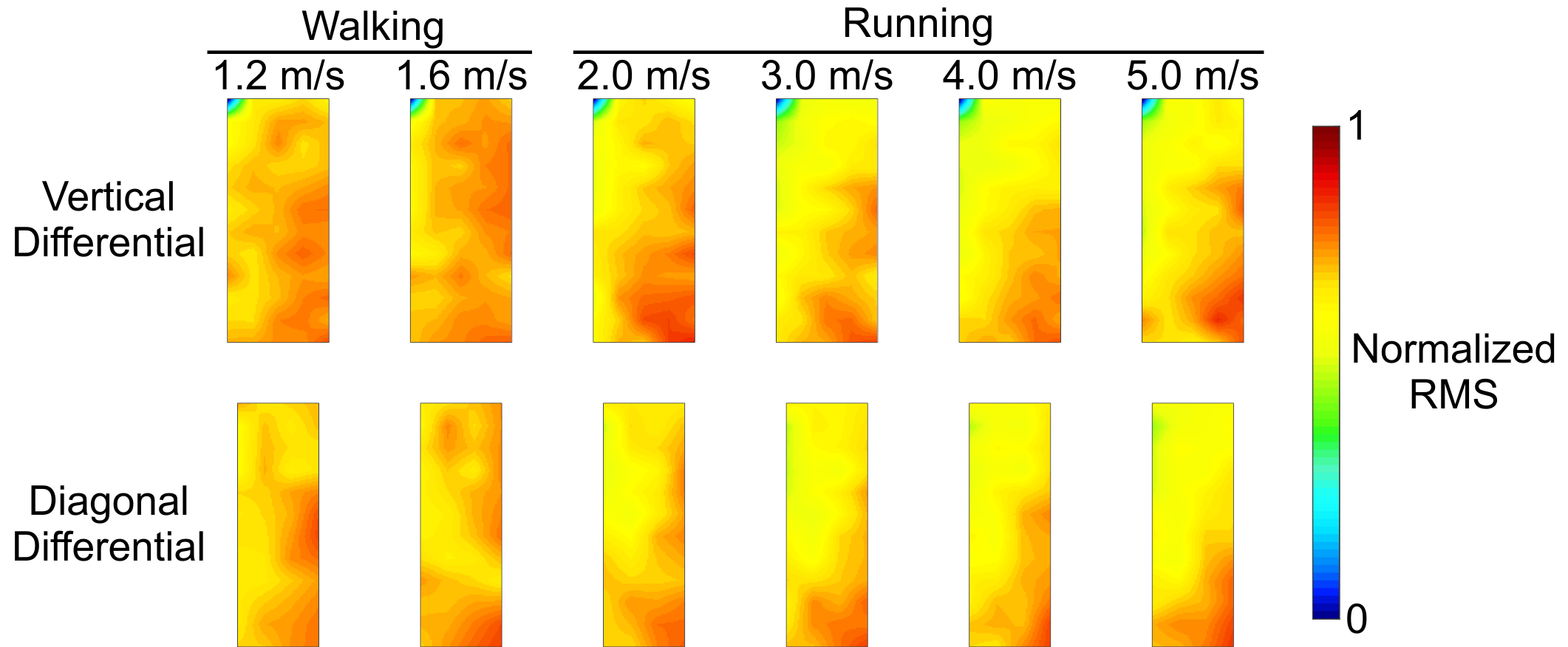

Supplement: Supplementary file 2 — Fig S2 [file PHY2-8-e14652-s002.tif]
